# Extensive novel diversity and phenotypic associations in the dromedary camel microbiome are revealed through deep metagenomics and machine learning

**DOI:** 10.1371/journal.pone.0328194

**Published:** 2025-07-17

**Authors:** Fathi A. Mubaraki

**Affiliations:** Faculty of Computers and Information Technology, University of Tabuk, Tabuk, Saudi Arabia; Kerman University of Medical Sciences, IRAN, ISLAMIC REPUBLIC OF

## Abstract

The dromedary camel, also known as one-humped camel or Arabian camel, is iconic and economically important to Arabian society. Its contemporary importance in commerce and transportation, along with the historical and modern use of its milk and meat products for dietary health and wellness, make it an ideal subject for scientific scrutiny. The gut microbiome has recently been associated with numerous aspects of health, diet, lifestyle, and disease in livestock and humans alike, as well as serving as an exploratory and diagnostic marker of many physical characteristics. Our initial pilot analysis of 55 camel gut microbiomes from the Fathi Camel Microbiome Project uses deep metagenomic shotgun sequencing to reveal substantial novel species-level microbial diversity, for which we have generated an extensive catalog of prokaryotic metagenome-assembled microorganisms (MAGs) as a foundational microbial reference database for future comparative analysis. Exploratory correlation analysis shows substantial correlation structure among the collected subject-level metadata, including physical characteristics. Machine learning using these novel microbial markers, as well as statistical testing, demonstrates strong predictive performance of microbial taxa to distinguish between multiple dietary and lifestyle characteristics of dromedary camels. We present strongly predictive machine learning models for camel age, diet (especially wheat intake), and level of captivity. These findings and resources represent substantial strides toward understanding the camel microbiome and pave the way for a deeper understanding of the nuanced factors that shape camel health.

## Introduction

The gut microbiome has been recently subjected to intensive study, and is found to be associated with numerous aspects of host physiology, including health and disease in humans and many domestic animals. These associations have improved our understanding of the interplay between environment and host in the context of outcomes of interest, and have resulted in biomarkers for conditions as varied as diseases and behavioral traits. In the context of agriculturally significant livestock, the microbiome may provide valuable insights into the animal well-being, which may have significant downstream economic implications [1–3]. As demonstrated by the Human Microbiome Project (HMP), which extensively cataloged the microbial populations within and upon numerous body sites of healthy humans, substantial differences in microbial taxonomy and population structure exist even within healthy individuals [4]. Notably, each body site exhibits distinct functional repertoires and ecological properties, meriting further study of specific body habitats for their respective contributions to overall host physiology. For example, each body site has its own natural diversity of microbes within it (alpha diversity), which in turn may be altered dramatically in response to health, environment, or lifestyle factors. The degree to which different microbiomes differ with respect to their microbial composition (beta diversity) also reveals ecological trends among hosts. This diversity, along with the taxonomic composition of the microbiome and its predicted functional capacity, have been shown to be associated with host health and environmental conditions [5]. The apparent host-specificity of the microbiome, particularly in the gut where diversity is high, along with its responsiveness to various conditions, suggests both descriptive and predictive potential of the microbiome, as well as a path toward individualized approaches to managing health and disease in livestock.

The role of the diet in shaping the microbiome of ruminant livestock has recently been explored. One recent study by De Menezes *et al*. highlighted significant differences in the rumen microbiome of dairy cows between cows that were fed via pasture grazing as opposed to a total mixed ration diet. Although this shows a clear influence of diet on one aspect of the gastrointestinal microbiome, the hindgut (fecal) microbiome was not investigated [6]. In another study, Tapio *et al*. present complex microbiome alterations induced by different forage:concentrate ratios with or without sunflower oil supplementation in dairy cows, revealing diet-specific shifts in diversity, taxonomic composition, and microbial co-occurrence patterns [7]. The implications of these shifts highlight the potential utility of the microbiome as a reporter or biomarker for the effects of diet and the environment, with practical ramifications for agricultural practices and animal nutrition. A better understanding of these associations may in turn enable detection of anomalies (such as disease) and optimization of feeding strategies to improve livestock health and productivity.

Although the rumen in true ruminants is anatomically distinct from the pseudo-ruminant foregut [8] of the dromedary camel, they play similar functional roles as primary degraders of complex plant material via microbial symbiosis in the upper digestive tract. A broad survey of the rumen microbiome across animal species [9], although lacking camels, includes microbiome samples from the rumens of pseudo-ruminant camelids due to this functional similarity, and additionally highlights the substantial role played by diet in shaping the microbiome of the rumen across all species studied. The effect size of dietary association was found to be larger than that of all other host factors measured. This close diet-microbiome relationship in the rumen is justified considering the rumen’s specialization in primary fermentative microbial digestion of complex plant material.

The metagenomic rumen microbiome of dromedary camels has itself been studied previously in at least two instances with shotgun sequencing. In one study of 3 dromedary camels that underwent shotgun metagenomic sequencing (7.7Gbp per sample), Gharechahi and Salekdeh [10] demonstrate the potential of metagenomic shotgun sequencing to analyze these poorly characterized communities, including limited genome reconstruction and carbohydrate-active enzyme (CAZyme) characterization using DNA assemblies. A high number of CAZyme annotations was found, as might be expected of microbiota specializing in complex plant fiber degradation. The authors also note the biases and limitations of previous studies using only 16S rRNA gene amplicon sequencing. In another broader study [11] of 48 rumen samples which underwent shotgun metagenomic sequencing (<2.47Gbp per sample), Hinsu *et al*. recapitulate the major finding of the aforementioned broad survey of rumen microbiomes [9] in dromedary camels; namely, that diet is a primary driver of the rumen microbiome composition. The study also confirms a high proportion of CAZymes in the shotgun sequencing data like Gharechahi and Salekdeh. Interestingly, Hinsu *et al*. go on to note the high prevalence of novel lineages of microbes detected, both in terms of uncharacterized genera identified by placeholders in existing databases, as well as novel genera that lack any representative genomes.

However, despite these early efforts to characterize the rumen microbiome of dromedary camels, the fecal microbiome, in contrast, remains largely unstudied at the resolution of shotgun metagenomic sequencing apart from a single older study by Dande *et al*. [12] of two pooled fecal samples sequenced at extremely low sequencing depth (<0.03 Gbp per sample). In that study, a total of 2 fecal samples were sequenced, not corresponding to individual camels but rather a pooled conglomerate of 2 camels each. This small sample size, low shotgun sequencing depth, and inability to distinguish subject-level microbiota due to the pooling process, all make it difficult to infer a statistical baseline against which to directly compare subsequent studies. Indeed, all of the camel metagenomic studies mentioned, including those studying the rumen, suffer from severe limitations in sample size, which preclude robust statistical comparisons due to lack of power.

Furthermore, it was also difficult to locate any study directly comparing the rumen to the fecal metagenome in dromedary camels, which might have otherwise established the extent to which studies of the rumen microbiome could generalize to the feces. However, some hints exist in the microbiome of a proximal species. The Bactrian camel, Camelus bactrianus, known for its distinctive pair of dorsal humps, has been the subject of microbiome research where the rumen and the fecal microbiome, along with multiple other regions along the digestive tract, were directly compared [13] using 16S amplicon sequencing. This effort revealed remarkable differences in microbial communities between the rumen and the stool, where the rumen had the lowest concordance with the colon/feces among all other gastrointestinal regions with which it was compared (abundance correlation of approximately 0.43 within animal at the genus level), along with a significantly higher alpha diversity than the feces. Taken together, this highlights the lack of readily comparable previous work in the fecal shotgun metagenomics of dromedary camels. Furthermore, amplicon-based studies (16S rRNA) have limitations in taxonomic resolution, as well as potential microbial abundance biases, as they focus on a single hypervariable 16S region, potentially providing a substantively incomplete picture of the gastrointestinal microbiota, limiting direct comparisons to shotgun metagenomics especially in cases where a substantial proportion of the community is novel [10, 14].

Likewise, few microbiome studies in camels have attempted to cross over into machine learning. Methodologically, while machine learning has contributed to measurable improvements in data analysis across various fields, its application to microbiome research has been largely concentrated on human subjects [15]. This leaves a significant gap in the understanding of animal microbiomes. One study demonstrated the utility of machine learning by training several models to predict colonic diseases using human microbiomes [16]. Machine learning models trained on fecal 16S rRNA [17] and metagenomic shotgun [18] data have shown significant potential in predicting diseases such as colorectal cancer. Another study employed metagenomic sequencing to probe the relationship between the gut genes of premature infants and their survival strategies in response to specific clinical and environmental conditions, revealing that formula feeding correlates with an increased presence of certain antibiotic resistance genes in the infant gut microbiome [19]. These examples underscore the potential of machine learning to reveal patterns that might not be discernible through traditional univariate statistical analyses, and hence may be important for better understanding the microbiome’s associations with health and other phenotypes. Therefore, we aim to bring these tools to the study of the dromedary camel as well.

This study aims to provide a comprehensive characterization of the microbial composition of dromedary camels, advancing the current understanding of the microbial diversity in the camel hindgut. By combining deep metagenomic shotgun sequencing, data mining, statistical analysis, and machine learning, we explore the camel microbiome and its host associations more comprehensively than previous work. This integrative approach is expected to pave the way toward an appreciation of the numerous factors influencing camel wellbeing and, by extension, the welfare of the human communities, economies, and ecosystems that depend on them.

## Materials and methods

### Ethics statement

This research was conducted in accordance with ethical standards set by the Research Ethics Committee at the University of Tabuk. Approval for the study was granted by the Research Ethics Committee under approval number UT-347-177-2022. Site owners (and farm staff, if the site was a farm) supervised all sampling activities. Governmental and local ordinances in Tabuk, Saudi Arabia do not require additional approval for passive collection and investigation of animal excrement from the ground.

### Camel fecal specimen collection and handling

Freshly excreted stool was sampled from 55 camels from the Tabuk region of Saudi Arabia from a variety of lifestyles and regional topographies. One fecal sample was collected from one fecal deposit from each individual animal. In brief, the collection protocol consisted of waiting until a camel dropped feces onto the ground, identifying the largest pellet without visible sand or dust on its surface, then immediately collecting the clean inner portion of the freshly passed stool with forceps and suspending 0.5-0.7g of material into 99% ethanol buffer (to preserve the specimen and prevent microbial growth [20]) within a pre-labeled specimen tube. Sampling was performed under supervision of local site managers. 27 distinct herds of camels were selected for our investigation, and at most 4 camels were sampled per herd (avg 2.04/herd). Camels were not interacted with during the sampling process.

### Sample storage and processing

Samples were stored under -20 C refrigeration while collection took place over a period of 30 days. Samples were shipped to BGI Hong Kong for DNA extraction and sequencing using the standard BGI complete DNA microbiome extraction kit. The extracted DNA was then sequenced using the DNBseq platform at 2 x 150 bp at 55 M sequencing pair read depth (110 M total reads per sample). Data was transferred via AWS S3 from BGI to a 64-core AMD Ryzen Threadripper Pro 5995WX server with 1.5 terabytes of RAM for downstream analysis.

### Raw data analysis

The metagenomics workflow from sample receipt, to extraction, to library prep and QC, sequencing, and read QC, was performed by BGI as part of their standard metagenomic shotgun sequencing offering. We also ran SHI7 quality control to confirm that the quality control performed by the provider was sufficiently high, indeed resulting in less than 0.2% additional read filtering in the worst case (sample 31 A), with average read length greater than 149.5 bp [21]. Host read filtering was not performed, as there were no concerns around de-identification, and minimal concern about reads mapping to contaminated reference genomes, both due to use of a custom database (see below) and the use of genome coverage thresholds in filtering the relative abundance table (see statistical and machine learning analysis methods below). Both single-sample and pooled assemblies were performed; in order to combine data from both methods, species-level representative genomes from the pooled assembly were only retained if a genome of the same species was not assembled from any single-sample assembly. Assembly was performed using megahit v1.2.9 [22]. MAGs were identified and binned using metabat2, and quality was assessed using CheckM2 v1.0.2 [23]. MAGs with assessed completeness >50% and contamination ≤5% were retained and clustered at 95% ANI in R from a distance matrix formed using aKronyMer v1.0 [24] using ANI GC LOCAL distance parameters with k-mer size 13. Representatives were selected from each cluster on the basis of highest aggregate completeness and lowest contamination (maximizing a score defined as (completeness−(5×contamination))).

Taxonomy was assigned with GTDB-tk v2.3.0 with GTDB R214 [25]. As most MAGs were unable to be placed within existing species-level designations, we opted to use a MAG profiling approach rather than existing microbiome databases used by popular tools which lack these novel species. Therefore, the resulting set of MAGs was used to create an XTree database for downstream analysis and profiling [26]. Read counts and unique coverage profiles were generated by XTree for all 55 samples, and the resulting representative species-level profiles were compiled into a species-level taxonomy table. An average of 74% of reads were able to be mapped back to the resulting MAG database. To assign a unique species-level placeholder name when GTDB was unable to assign a reference species name, we used an arbitrary genome ID as a placeholder species name. Genus-level aggregation was also performed to assign a consistent placeholder name to genus-level representatives from species that clustered together at ≥90% ANI (and <95%) using the same approach outlined above.

### Statistical and machine learning analysis

Correlations were performed in R (v4.3.0). Species richness was computed by summing the number of non-zero species per sample, defining a genome as present when its genome is greater than 25% uniquely covered in that sample. Beta diversity was calculated using R’s cmdscale function on log10-scaled species relative abundances (log euclidean). Bray-Curtis dissimilarity was also used for comparison. A machine learning model was fitted with the randomForest package in R (using 5000 trees and default parameters without hyperparameter tuning), using the relative abundance data to predict covariates in the metadata, including dietary and lifestyle features. Reported ML performance scores (AUC for binary features) were calculated using random forest out-of-bag predictions; feature importance was assessed using the Gini index. For increased generalizability in this 55-sample dataset, genus-level taxonomy was used for all machine learning and differential analysis.

## Results

The Fathi Camel Microbiome Project resulted in the collection and metagenomic sequencing of fecal material from 55 camels, along with a number of metadata fields (covariates) per camel. This allowed for the generation of a robust database of metagenome-assembled genomes (MAGs), as well as the ability to perform statistical associations.

### Demographic summary highlights the diversity of sampling

To reflect potential metagenomic diversity in camel microbiomes, we sampled from a diverse set of camels from the Tabuk region of Saudi Arabia. The demographic summary of animal characteristics is presented in Table 1 below, highlighting a diversity of ages, diets, and habitats of these 55 camels across 27 herds.

**Table 1:** Demographic characteristics of the FCMP, separated into numerical variables (A) and categorical variables (B). Coding was determined as specified in (C). More collected variables are provided in the supplementary metadata table.

(A) Numerical Variables						
Variable (num)	Min	Q1	Median	Mean	Q3	Max
Age (y)	0.3	4	7	6.01	8	12
Weight_Factor	1	3	3	3.02	3	5
Diet_Diversity	1	1	2	1.73	2	4
Bristol_Factor	1	3	4	3.64	4	7
Species_Richness	74	435.5	498	478.22	548	619
(B) Categorical Variables						
**Variable (Cat)**		**Category**			**Count**	
Gender		F			45	
Gender		M			10	
Pregnancy		No			37	
Pregnancy		Yes			18	
Disease_Status		Healthy			49	
Disease_Status		Sick			6	
Living_Condition		auction			24	
Living_Condition		farm			16	
Living_Condition		wild/farm			15	
(C) Coding Notes						
**Variable**			**Coding notes**			
Weight_Factor			Qualitative observation of camel weight, accounting for age and body fat. 1 = emaciated, 5 = obese			
Diet_Diversity			Sum of the number of distinct dietary streams provided to the camel within the last 3 months. Different dietary components measured are: grass, wheat, barley, bread, milk, vitamin supplement. Ranges from 1 to 6 (but no camel has > 4).			
Bristol_Factor			Modified Bristol Stool Index (1-7)			
Species_Richness			Richness in number of (prokaryotic) species detected			
Disease_Status			Farm reported and investigator confirmed presence of physical or GI symptoms			
Living Condition Captivity_Factor			Living condition of auction, farm, or wild/farm (free-roaming herd) were factorized into 3, 2, and 1 captivity levels, respectively			

### A comprehensive prokaryotic genome database for camel microbiome analysis

**Fig 1:**
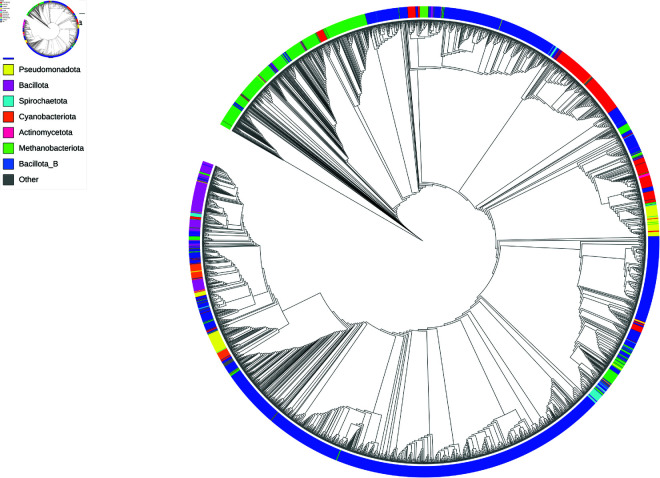
An extensive set of reference genomic MAGs spanning 3,165 new species-level representatives. Average nucleotide identity-based ladderized circular cladogram depicting approximate relationship among the genomes, generated by iTOL. Narrow, deeply-branched regions indicate phylogenetic singleton genomes, while wider clades near the edges indicate the presence of more closely-related species. The leaves are colored by phylum designation per GTDB-tk R214.

An important outcome of this effort was the generation of a prokaryotic reference genome database from direct assembly of deeply sequenced camel fecal metagenomes. A total of 3,165 species-level prokaryotic (bacterial and archaeal) genomes were produced with CheckM2 completeness ≥ 50% and contamination ≤ 5%, per standard MiMAG quality criteria [27]. After taxonomy assignment, 726 genera were found, with 55 containing > 10 species. Up to 151 genera were novel (as they contained no CheckM2 identification). 2,740 of the 3,165 species-level genomes (87%) represented novel species without any species-level database reference or representative genome in the GTDB. This high level of novelty is expected given the lack of deep metagenomic sequencing efforts in dromedary (Arabian) camels apart from the current study. As shown in Fig 1, the phylogenetic tree was visualized using iTOL [28].

On average, 74% of the raw reads were mappable back to the FCMP MAG database, indicating reasonable representation of the prokaryotic microbiome in this set of camels. Incidentally, a BLAST analysis using the NCBI nt database on a random subset of 100 contigs (of length under 200 kbp and low multiplicity of 1-2x coverage) not binned into MAGs revealed an assortment of unknown DNA (no matches with default or sensitive blastn parameters), plant matter from wheat (94–98% identity matches) and barley (~85% matches), unknown plant material (perhaps local desert grasses or shrubs; including a 70% match of <10% sequence length by blastn to various plants), and unknown eukaryotic DNA (including 2 matches with 75% identity to various insects). However, our analysis focuses on the prokaryotic members of the camel hindgut microbiome, and further analysis of the non-prokaryotic “dark matter” in the stool will be left to future work.

### Taxonomic visualization across the dataset

**Fig 2:**
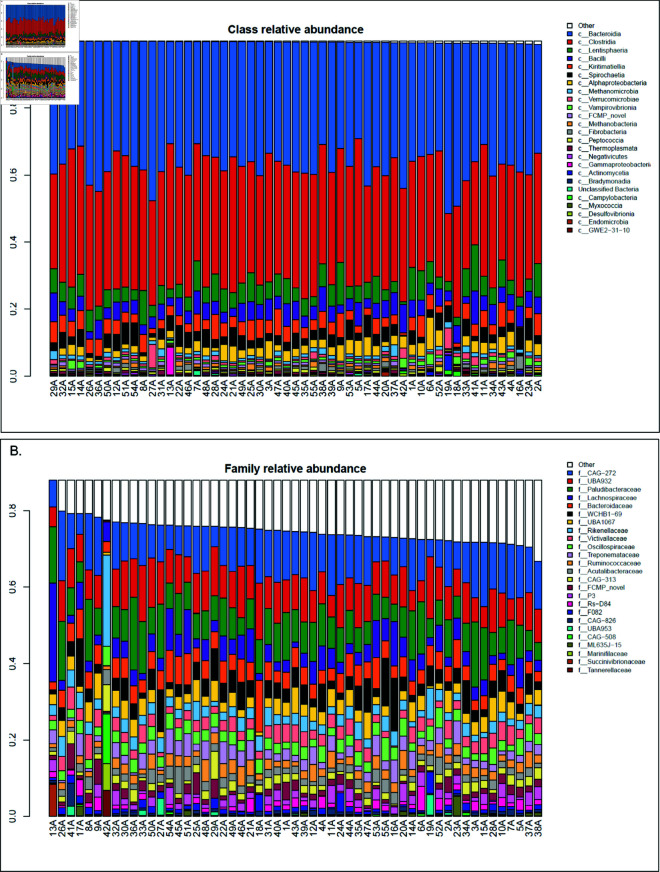
Class and family relative abundance across the dataset. (A) Class-level relative abundance, sorted by fraction of “Other" (classes not within the top 25 by abundance across the dataset). (B) Family-level relative abundance, sorted by decreasing fraction of “Other" taxa (taxa not within the top 25 by relative abundance across the dataset). Plots are truncated to the minimum level of unknown taxa for visualization purposes, hence the truncated y-axis.

To visually ascertain the degree to which microbes are prevalent and abundant across the dataset and the variability associated with them, we generated stacked barplots of each camel’s microbiome in the dataset, sorted by most abundant microbes, An aggregated summary of the prevalence and abundance of the novel taxa is presented in each figure as “FCMP novel”, representing all the novel taxa at a given taxonomic rank. At the class level, novel classes do not enter into the most abundant 25 classes in the dataset, so they are not displayed, [see Fig 2A]. At this level of taxonomic summarization, it is apparent that 70–75% of the dromedary camel microbiome by relative abundance is dominated by classes Bacteroidia and Clostridia. At the family level [see Fig 2B], we see novel families (indicated in maroon) appear throughout the dataset. The overall microbial composition at the family level is dominated by CAG-272, UBA932, Paludibacteraceae, Lachnospiraceae, and Bacteroidaceae.

**Fig 3:**
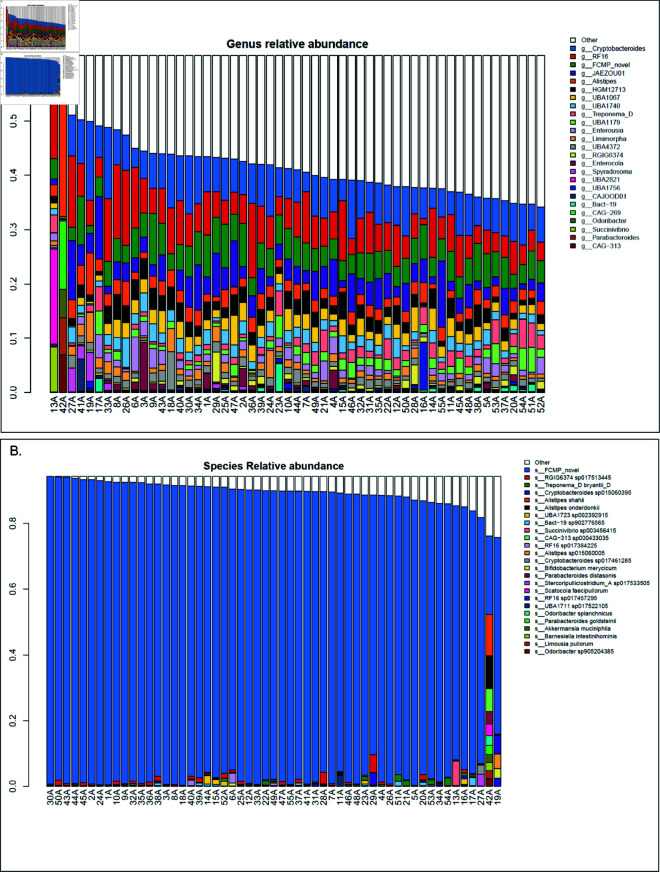
Genus and species level abundance across the dataset. (A, B) Genus-, and species-level relative abundance, each sorted by decreasing fraction of “Other” taxa (taxa not within the top 25 by relative abundance across the dataset). Plots are truncated to the minimum level of unknown taxa for visualization purposes, hence the truncated y-axis.

At the genus level [see Fig 3A], there is substantially more diversity than can be represented by the top 25 genera (the “Other” category, encompassing all genera that are not in the top 25, occupies the majority of most microbiomes), but here we see the novel genera discovered by the present study (in green) are the third most abundant members overall (following Cryptobacteroides and RF16). At the species level [see Fig 3B], it is apparent that novel species (in blue) occupy the majority of the microbiome. Notably, sample 42A is an extreme outlier here like it was in the beta diversity analysis. 42A is the youngest camel in the dataset at just 3 months of age versus the dataset average of 6 years, and the only camel exclusively breastfed. This difference becomes more distinct at finer taxonomic levels.

The present study’s finding of *Cryptobacteroides* (formerly a member of *Bacteroides*), *Alistipes*, and *Treponema* among the most abundant set of (named) genera in dromedary camel feces is consistent with the findings of [12], but the larger number of novel and uncharacterized genomes discovered here dominate the microbiome and highlight the importance of building out infrastructure to quantify the microbial novelty within the dromedary camel fecal microbiome. Indeed, most of the microbes discovered here are completely missed by the use of older, limited databases consisting solely of well-known microbes.

### Associations between metadata and diversity

**Fig 4:**
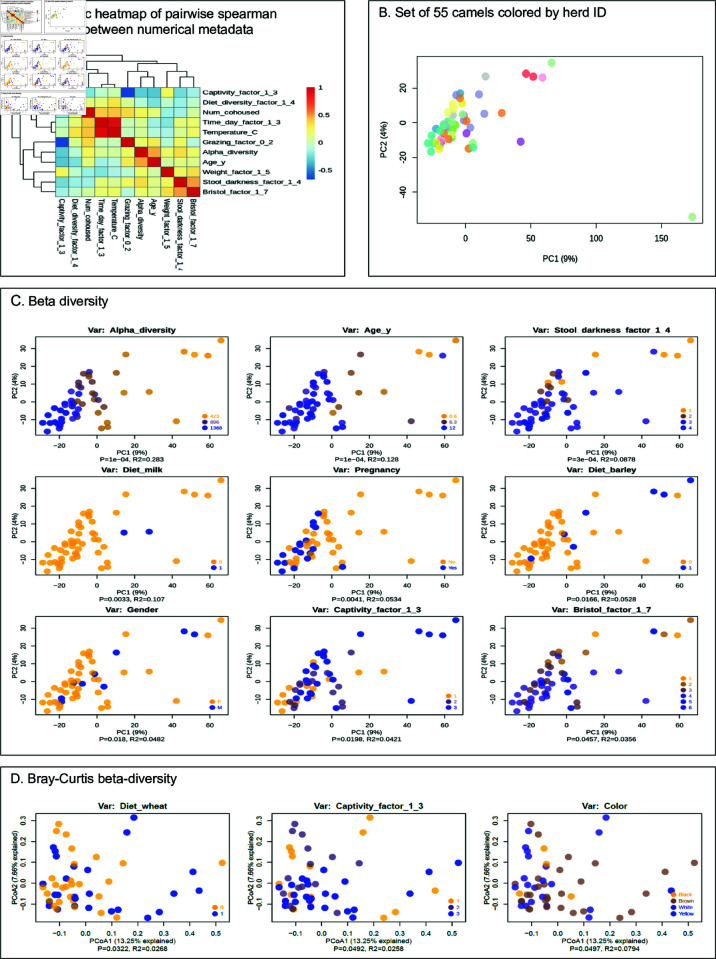
Principal components plots reveal beta diversity distribution colored by various metadata variables (covariates). (A) Symmetric heatmap of pairwise spearman correlations between numerical metadata. (B) shows the entire set of 55 camels colored by herd ID. The outlier in the bottom right is from a camel that was in its third month of life, and was removed (for plotting purposes only) for subsequent beta diversity plots in (C). (C) Beta diversity (log euclidean distance) colored by significantly associated covariates as determined by PERMANOVA p < 0.05. ${\text{R}}^{2}$ value (effect size of association) is also displayed under the x axis along with the p-value of the association. For continuous variables, the legend shows the minimum, maximum, and midpoint of the distribution of values plotted. Percentages in axes labels are in terms of percent variance expressed by each axis. The points are in the same place in all plots; only the colors and statistical results differ by metadata variable. (D) Similar to (C) but using Bray-Curtis beta-diversity and the significant associations found using this metric.

A pairwise all-vs-all spearman correlation among continuous variables showed some expected correlations between collection-related variables, such as time of day and temperature, [see Fig 4A]. Some biological variables also clustered by correlation, such as Bristol index (stool consistency) with stool darkness in one cluster, and species richness with age in another. As expected, level of captivity inversely correlated with the amount of grazing reported, as well as species richness and age (younger camels are more likely to be kept in controlled auction environments).

A visual inspection of the herd-labeled beta diversity ordination, [see Fig 4B], appears to confirm the expectation that camels from the same herd have more similar microbiomes, with a close clustering of like-colored points. A clear outlier microbiome sample is also apparent in the same plot, which was collected from a very young calf (in its third month of life) which was exclusively breastfeeding, unlike any other camel in the dataset (the next youngest is twice its age and consuming solid food).

We tested each metadata variable in the binary and numerical sets using PERMANOVA on the beta diversity distance matrix to determine the significance of the association, and displayed all significant results as colored annotations in [see Fig 4C]. Notably, species richness was a significant driver of beta diversity, forming a clear gradient along PC1, and age followed a similar trajectory (age is also associated with species richness directly; spearman r = 0.61, p = 7.4e-7; pearson rho = 0.48, p = 0.0002). Bristol index and stool darkness followed similar gradients along PC1.

Captivity factor (the degree of captivity), however, exhibits a reverse relationship. Of note, although dietary diversity does not produce a significant association, individual dietary components (milk, barley) show some evidence of microbiome clustering. Pregnancy status and gender also produce significant separation. Notably absent from the significantly associated beta diversity results are disease status (we return to this point in later analyses), weight, and number of co-housed camels. We also evaluated Bray-Curtis dissimilarity in discriminating between camels by covariates, but found that it discriminated less well between microbiome samples with respect to metadata, with only 3 significant associations found (wheat diet, captivity status, and color) [see Fig 4D].

### Differential analysis and machine learning

**Fig 5:**
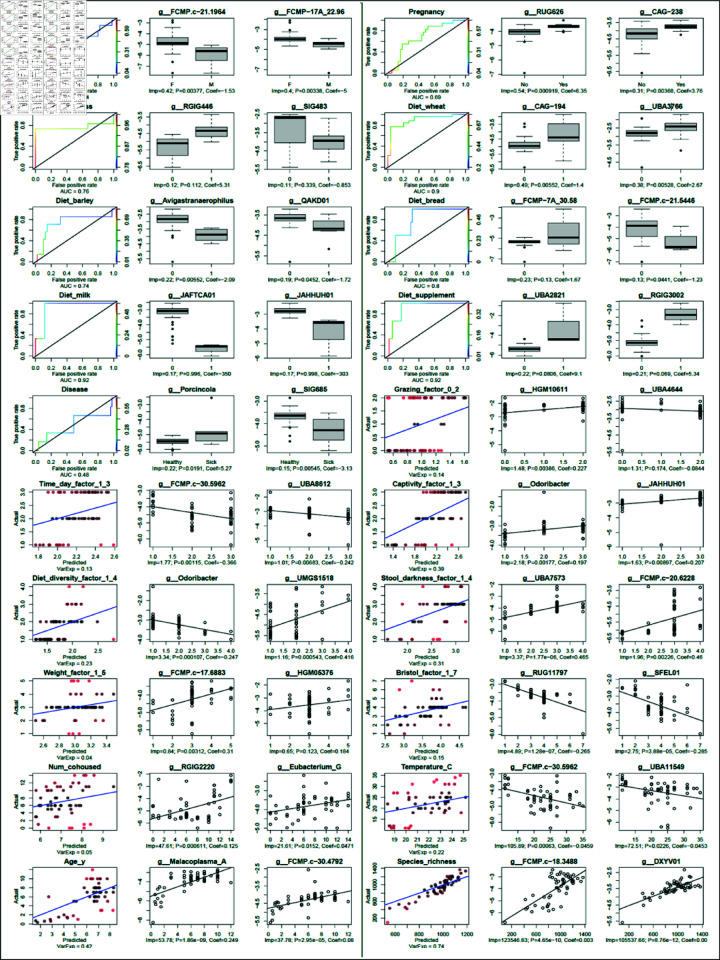
Visual summary of machine learning and statistical association testing. The left-most figure in each panel shows the random forest performance of a model trained on the given variable. Binary variables were used to run random forest models in classification mode, whose performance is conveyed using a colored Receiver Operator Characteristic (ROC) curve, colored by the class probability threshold used for predicting class membership and labeled for the predictive performance based on Area Under the Curve (AUC). The identity (y=x) function has been added, denoting the performance of a hypothetical random model (AUC = 0.5). Continuous and ordinal variables were subjected to classification by random forest, which is shown as a scatterplot of the actual value against the out of bag prediction. The identity (y=x) line has been added, denoting hypothetically perfect predictive performance (all predicted values exactly match the true values). Points are colored by their distance from the identify function; more intense red signifies poorer prediction for those values. To the right of each machine learning performance summary plot are two plots showing the log10 relative abundance of the top 2 genera (highest Gini index for the random forest), as either boxplots (classification) or univariate scatterplots (regression) of the genus vs the variable. Each univariate genus plot is labeled with the Gini importance score (“Imp”), as well as both the p-value and coefficient from a univariate model fit (logistic model for binary variables, linear models for the rest).

Relative abundances of genera were used as features to train a random forest model across all metadata variables, [see Fig 5]. Additionally, the top-scoring genera by feature importance were also separately visualized and analyzed using univariate regression and associated statistics. Strikingly, some variables, including all binary dietary features, yielded strong predictive performance (OOB ROC AUC > 0.72), including some features that were not significantly distinguished by earlier analyses of diversity metrics (community-wide), or only with specific beta diversity metrics. This is especially apparent with the prediction of dietary wheat, which produced a non-significant association with beta diversity using log-euclidean distances (PERMANOVA p = 0.13) and a weakly significant association using Bray-Curtis (p = 0.032), yet a 0.9 AUC value by random forest prediction using genera relative abundance. Considering the wheat feature in particular is fairly class-balanced (29 camels are not wheat consumers, and 26 are), this predictive performance is less likely to be artifactual than other results with less balanced classes such as milk or grass, where only 3 camels did and didn’t consume these dietary sources, respectively. Milk and grass consumption are also confounded by age and wheat consumption, both of which are represented by highly predictive models.

The biological interpretation of these results can be challenging, particularly where the taxonomy is poorly-defined, but insights emerge regardless. The top genera predicting wheat consumption, *CAG-194* and *UBA3766*, are both from family Lachnospiraceae, a family which has been shown to be associated with wheat diets in dogs [29]. Comparatively little is known about the role of *Candidatus Avigastranaerophilus*, a genus which is depleted in camels eating barley diets, other than that it was initially discovered in chickens [30]. As another example, the association of a *Malacoplasma* genus (of family Mycoplasmoidaceae) with age, particularly within the first 3 years of life, is notable if a bit unexpected. One possible explanation is that the fermentative capacity of young camels may be developing rapidly in the first few years of life, and this microbe may increase in abundance alongside the development of the young camel’s fermentative capacity, feeding off of the fermentation process more effectively over time (some adjacent clades like *Mycoplasma* contain well-known saprotrophs). Genus *Odoribacter*’s dual top associations – positively with captivity and negatively with dietary diversity, make sense in context of the (weak) negative correlation between these two factors themselves. *Odoribacter* is known to be associated with dietary and environmental factors in humans [31], and might plausibly rise in conjunction with a more controlled and potentially less diverse diet in captivity.

### Univariate nonparametric statistical association testing

In addition to machine-learning-based random forest modeling, classical non-parametric statistical tests (spearman rank correlation, Wilcoxon rank-sum test) were conducted [see Table 2] to evaluate the univariate association of each microbial genus with each metadata outcome variable, which was followed by multiple hypothesis testing correction using FDR. Notably, the *Bifidobacterium* genus was found to be positively associated with dietary diversity (number of distinct food sources in a camel’s diet), a number of uncharacterized (but GTDB-recognized) genera were negatively associated with the Bristol stool scale, and a number of novel genera (FCMP prefix) were found to be associated with camel age. Many results recapitulate the features found most important in the random forest model. For example, genus FCMP.c-21.1964, a member of Gastranaerophilales family RUG14156, was found to be more abundant in female camels in both approaches. Likewise, RUG626, a member of family Oscillospiraceae, was found to be associated with pregnancy, and FCMP.c-30.4792, a member of Clostridia order TANB77 family UBA1234, was found to be associated with age, and so on. But some of the top organisms do not perfectly align between the two methods. For instance, the top 2 predictors of diet diversity in the machine learning model do not overlap with the 2 most associated genera by univariate testing. Univariate association testing also revealed a possible slight depletion of genus FCMP-50A 17.82 of family Peptococcaceae in diseased camels (FDR p = 0.077), despite a lack of predictive ability in the machine learning model of the same.

**Table 2:** Univariate association testing reveals genera associated with various outcomes (top associations). Spearman correlation was used for continuous variables, and Wilcoxon rank-sum tests were used for binary variables. P values were adjusted after all testing using Benjamini-Hochberg FDR. Variables in italic indicate binary variables (Gender, Pregnancy, Diet_barley, Diet_wheat). The Direction column indicates for continuous variables (or ordered factors) whether the genus relative abundance is positively (+) or inversely (-) correlated with the outcome variable, and for binary variables displays which outcome has the higher relative abundance (e.g., “Yes" means the genus is higher in the “Yes" class). Magnitude indicates rho (Spearman correlation coefficient; for continuous variables) or log10 fold change between the two classes (for binary variables).

Top genera associations				
Genus	Variable	adj_P_Value	Direction	Magnitude
g__UBA6857	Bristol_factor_1_7	0.001	-	-0.647
g__Mailhella	Captivity_factor_1_3	0.002	+	0.627
g__JALENY01	Bristol_factor_1_7	0.002	-	-0.617
g__Bifidobacterium	Diet_diversity_factor_1_4	0.002	+	0.617
g__HGM12650	Stool_darkness_factor_1_4	0.002	+	0.614
g__CALVUJ01	Age_y	0.002	+	0.610
g__JAHHUH01	Captivity_factor_1_3	0.002	+	0.602
g__SFTH01	Bristol_factor_1_7	0.005	-	-0.581
g__UBA738	Bristol_factor_1_7	0.005	-	-0.580
g__RGIG8767	Bristol_factor_1_7	0.005	-	-0.580
g__Enterocola	Age_y	0.005	+	0.577
g__HGM12713	Captivity_factor_1_3	0.005	-	-0.574
g__UMGS1865	Captivity_factor_1_3	0.007	-	-0.563
g__Phocaeicola	Captivity_factor_1_3	0.007	+	0.562
g__W1P20-047	Bristol_factor_1_7	0.007	-	-0.560
g__Caccosoma	Age_y	0.007	+	0.558
g__Dysosmobacter	Captivity_factor_1_3	0.007	+	0.557
g__FCMP.c-30.4792	Age_y	0.007	+	0.555
g__FCMP.c-21.1964	Gender	0.007	F	1.162
g__RUG626	Pregnancy	0.007	Yes	0.415
g__UBA7573	Stool_darkness_factor_1_4	0.008	+	0.551
g__RUG11797	Bristol_factor_1_7	0.009	-	-0.549
g__Avigastranaerophilus	Diet_barley	0.009	0	1.013
g__RGIG1899	Captivity_factor_1_3	0.009	+	0.545
g__FCMP.c-27.6331	Age_y	0.009	+	0.543
g__UBA7862	Age_y	0.009	+	0.543
g__FCMP.c-16.5802	Age_y	0.009	+	0.541
g__FCMP.c-20.6228	Stool_darkness_factor_1_4	0.009	+	0.541
g__UBA71	Captivity_factor_1_3	0.009	+	0.541
g__Dysosmobacter	Bristol_factor_1_7	0.009	-	-0.540
g__RGIG3947	Age_y	0.009	+	0.540
g__CAKVLS01	Age_y	0.010	+	0.539
g__CAG-269	Diet_diversity_factor_1_4	0.010	-	-0.537
g__Malacoplasma_A	Age_y	0.010	+	0.535
g__FCMP.c-25.3551	Stool_darkness_factor_1_4	0.010	+	0.535
g__FCMP.c-20.6228	Bristol_factor_1_7	0.011	+	0.532
g__RGIG3994	Stool_darkness_factor_1_4	0.012	+	0.529
g__Spyradomonas	Diet_barley	0.012	0	0.795
g__RGIG3091	Captivity_factor_1_3	0.012	-	-0.527
g__HGM13010	Temperature_C	0.012	-	-0.527
g__UBA3210	Age_y	0.012	+	0.526
g__Acetatifactor	Bristol_factor_1_7	0.012	-	-0.526
g__Zag111	Captivity_factor_1_3	0.013	-	-0.524
g__UBA1731	Stool_darkness_factor_1_4	0.013	+	0.523
g__UBA1425	Bristol_factor_1_7	0.015	-	-0.520
g__FCMP.c-30.2690	Age_y	0.015	+	0.518
g__UMGS1384	Bristol_factor_1_7	0.016	-	-0.517
g__FCMP.c-22.8530	Captivity_factor_1_3	0.016	-	-0.516
g__RUG11130	Temperature_C	0.016	-	-0.516
g__Egerieousia	Captivity_factor_1_3	0.016	+	0.515
g__UBA3766	Diet_wheat	0.016	1	0.400

## Discussion

The Fathi Camel Microbiome Project pilot provides powerful early insights into the diversity and ecological drivers of the dromedary (Arabian) camel microbiome, and lays the groundwork for subsequent analyses and expansion. Although some significant limitations exist at this stage, such as small sample size and limited geographical range of sampling, the study design compensates for lack of breadth with greater depth: the use of ultra-deep whole-metagenome shotgun at 110 million total read depth per sample is unprecedented in the literature for the study of camels and most other livestock. Accordingly, this work adopts a metagenomic analysis approach more common in human metagenome studies. Furthermore, there is considerable potential for the genomic reference databases generated here to more comprehensively characterize future (including shallower) metagenomic studies in camels, as alignment of metagenomic data against an existing reference database (such as that produced by this study) can substantially reduce the barrier to entry for future work in this agriculturally and culturally important area.

Other limitations include the potential for environmental contamination, a risk inherent in the study of animal fecal communities. This risk was somewhat mitigated by sampling only the inner portion of freshly dropped fecal material, devoid of visible sand and dust. Another limitation is using out-of-bag prediction performance reporting for our random forest models, rather than an explicit cross-validation framework. This is largely due to sample size constraints. Random forests have been previously reported to work well in the high-dimensional low-sample-size domain for microbiome data [32], and out-of-bag prediction performance, which while often representative, may in some cases be optimistic or inaccurate. Multiple steps were taken to mitigate overfitting risk, including using 5000 individual trees in the random forest ensemble (increasing the diversity of decision trees to average out bias and stabilize test error), avoiding hyperparameter tuning, not comparing multiple models in pursuit of any “best" model, and most importantly, including a secondary, independent statistical validation that adds confidence in the results using classical univariate tests with p-values. Nevertheless, the purpose of this approach as implemented in this study is not to create a robust production model, but as a proof of concept to highlight areas of biological interest warranting future follow-up and secondary validation with future sampling efforts.

Some of the strongest associations reported in this analysis relate to age and diet, two interleaving aspects of development that have been shown to be correlated in humans as well [33, 34]. The ability of the microbiome to strongly predict (or strongly associate with) dietary components in our study, most notably wheat, speaks to the ecological ramifications of microbe-food associations writ large. Is it a commensal microorganism that aids digestion of wheat fiber specifically? Or a common plant symbiont merely passing through? Such questions would benefit from longitudinal analysis, interventional studies using dietary swaps, or thorough environmental sequencing of the foodstuff (e.g. plant material) to address these open questions.

Just as notable, but perhaps not unexpected, is the lack of meaningful predictive ability for disease status given microbiome signatures (although there was a weak statistical correlation). Although the microbiome has been implicated in numerous diseases in humans and livestock, we remain underpowered to detect disease trends in this study due largely to the lack of diseased camels in the populations visited at time of sampling, as well as more detailed information on the disease etiology for the few diseased camels that were sampled. Without finer-grain control of the diseases sampled, detection of disease signals may be limited to general dysbiosis signatures, which may require significantly larger numbers of matched diseased and healthy camels for sufficient discriminatory power.

## Future directions

In terms of project scope and expansion, our near-term goal is to expand the sample number to 200-500 camels, as well as add targeted sequencing of camel milk, which is of vital social and economic significance to the region. We are also planning to sample a larger (or second) geographical area and explicitly address the question of whether Arabian camel microbiomes have strong geography-specific taxonomic signatures.

Analytically, we are planning to perform cross-domain (viral, fungal, eukaryotic microbial) identification of microorganisms, and attempt to tag consumed plant matter in the fecal DNA. With expanded geographical sampling, we hope to train models to better generalize geographically, and assess that generalization performance explicitly via a leave-one-region-out training approach. Analysis of the results of gene calling, protein clustering, and deep functional annotations of this deep metagenomic data would make attractive targets for future work (and indeed, we have already completed much of the computational work underlying this data, as it involves steps which were necessary for other steps in our Methods above; e.g. gene-calling and KEGG ortholog functional annotations are required inputs for CheckM2). Use of functional annotations may allow us to mechanistically describe the relationship between microbes and the dietary or environmental features with which they were found to be associated by taxonomy; this is also left to future work.

However, due to the high-p low-N dimensionality issues discussed above in the context of the small sample sizes available to us in this initial effort, further increasing the feature space (with genes, proteins, viruses, etc) was deemed disadvantageous without a concomitant increase in sample number, which is planned for the next stage of work for the FCMP.

## Conclusions

Overall, our study provides an intensive investigation into the gut microbiome ecology of the Arabian camel. Utilizing deep metagenomic shotgun sequencing, we reveal a remarkable amount of novel microbial diversity within 55 sampled camel gut microbiomes. We develop a comprehensive, publicly available database and genomic resource for use in prokaryotic species identification using metagenome-assembled microorganisms. We hope that our microbiome reference database will prove to be a valuable resource for data analysis in camels, as well as in expanding the catalog of global microbial diversity discovered to date.

Our investigation of the correlations between the Arabian camel microbiome and metadata covariates exposed notable microbial trends with respect to physical features of these Arabian camels, as well as captured camel gut ecological diversity in broad strokes. Our study also provided a noteworthy examination into prospective predictive biomarkers for many of these variables, highlighting the potential to generalize and expand upon these with more samples, and potentially provide clues into the intimate relationship between camels, the microbes they host, the foods they consume, and the environment in which they live.

In conclusion, our study has made significant strides in deeply characterizing the species-level microbiome diversity in Arabian camels. It also holds the potential to further advance metagenomic studies in camels and beyond, and provides a valuable reference database with which to compare results across future studies. It uncovered thousands of novel prokaryotic microorganisms without species (or often even genus-level) representatives in existing databases, opening up new areas to explore in comparative genomics and systemetology. Most importantly, our study’s early insights and exploratory framework may pave the way for future work to further illuminate the previously unexplored microbial communities within these iconic mammals, including potential future insights into the health and productivity of dromedary camels and beyond.
